# Effects of Impurities from Sugar Excipient on Filtrate Flux during Ultrafiltration and Diafiltration Process

**DOI:** 10.3390/membranes11100775

**Published:** 2021-10-11

**Authors:** Jieun Lee, Jiwon Na, Youngbin Baek

**Affiliations:** Department of Biotechnology, Sungshin Women’s University, Seoul 01133, Korea; je_0229@nate.com (J.L.); qas111026@gmail.com (J.N.)

**Keywords:** sugar excipient, sucrose, formulation, filtrate flux, diafiltration, ultrafiltration, nanoparticulate impurities

## Abstract

Sugar excipients such as sucrose and maltose are widely used for biopharmaceutical formulation to improve protein stability and to ensure isotonicity for administration. However, according to recent literature, pharmaceutical-grade sucrose contained nanoparticulate impurities (NPIs) that result in protein aggregation and degradation. The objective of this study was to evaluate the filtrate flux behavior of sugar solution during ultrafiltration (UF) and diafiltration (DF). Filtrate flux data were obtained using either a tangential flow filtration (TFF) system for DF experiments or a normal flow filtration system for UF experiments. In diafiltration experiments, which were performed using 7 g/L of human immunoglobulin G in a 20 mM histidine buffer with the 100 mM sucrose or maltose, the filtrate flux with sucrose solution decreased significantly. In contrast, the one with maltose solution was in good correspondence with the calculated filtrate flux accounting for the effects of solution viscosity. This large decline in the flux was also observed during UF experiments, in which the presence of NPIs was identified by dynamic light scattering analysis and by capturing an SEM image of the membrane surface after filtration. In addition, highly purified sucrose resulted in a much lower flux decline in TFF in the absence of NPIs. These results provide important insights into the factors governing the optimization of the UF/DF process using appropriate excipients for biopharmaceutical formulation.

## 1. Introduction

Antibody-based therapeutics have recently become the predominant class of drugs, with more than 80 approved and commercialized monoclonal antibodies (mAbs), since muromonab-CD3 (i.e., Orthoclone OKT3 as the first therapeutic mAb) was approved in 1986 [1,2]. The global market for mAb therapeutics is predicted to approximate USD 319 billion by 2026 [3]. Among the advantages of mAbs are their high specificity and fewer adverse effects [4], which enable them to be successfully used for the treatment of various human diseases such as cancers, and autoimmune diseases such as rheumatoid arthritis and Crohn’s disease [5].

Bioprocessing to manufacture antibody-based therapeutics is composed of an upstream process for growing cells capable of producing the desired pharmaceutical compounds and a downstream process for protein separation/purification [6]. The downstream process, which is important to determine the efficacy and stability of biotherapeutics, accounts for 30–50% of the total production costs (for particular therapeutics, this could be as much as 70%) [7]. This process comprises the following steps: clarification, primary capture, polishing, virus filtration, and formulation [8]. The formulation process usually includes ultrafiltration (UF) and diafiltration (DF) for concentration and buffer exchange [9] in the order of UF-DF or UF-DF-UF, depending on the final concentration of proteins; the former is used to manufacture proteins at a relatively low concentration (i.e., a few tens of mg/mL) for intravenous injection, and the latter to manufacture highly concentrated proteins for subcutaneous injection [10]. However, highly concentrated protein solution has high viscosity, with a tendency to form aggregates in solution [11]. Thus, excipients which either induce changes in the conformational or colloidal stability of proteins in solution are used to preserve the colloidal stability and guarantee reliable processing and safe formulations [12].

Sucrose is a common sugar excipient for the antibody-based therapeutics (e.g., it is currently used for ENBREL^®^, XOLAIR^®^, and ILARIS^®^ [13]) during the formulation process, in order to improve the protein stability, and to reduce protein denaturation [11,14]. The ability of sucrose to improve the stability of proteins in solution can be understood by considering that water molecules are preferentially excluded from surrounding the proteins (i.e., sucrose molecules preferentially surround the proteins instead of water molecules) [15]. Nonreducing sugars (e.g., sucrose and trehalose) are also known not to participate in glycation with proteins [16]. Apart from this, the additional role of sucrose is to ensure isotonicity (i.e., ~290 mOsm/L), which serves to reduce the damage to red blood cells and tissues during injection [17]. Other than sucrose, maltose, NaCl, sorbitol, mannitol and trehalose have also been widely used as an excipient for biopharmaceutical formulation since 2010 [17].

However, following recent literatures, sucrose, even of pharmaceutical-grade quality, contained nanoparticulate impurities (NPIs) from 100–200 nm [18]. These NPIs give rise to signals that interfere with those of sugar-containing solutions, and were identified using dynamic light scattering and nanoparticle tracking analysis. The analyses suggested that the NPIs were agglomerates of various impurities, including dextran, ash, and aromatic colorants originating from raw materials. Furthermore, the NPIs have been shown to negatively affected the stability of mAbs, such as trastuzmab, riruximab, infliximab, and cetuximab [19]. The injection of NPIs purified from pharmaceutical-grade sucrose into the mAbs resulted in protein aggregation and degradation.

The objective of this study was to examine the effects of sugar excipient on the membrane during the UF/DF process. Filtrate flux data were obtained using either a tangential flow filtration (TFF) or a normal flow filtration (NFF) system. The filtrate flux behavior during diafiltration was compared using 7 g/L of human immunoglobulin G (IgG) in 20 mM histidine buffer with either 100 mM sucrose or maltose. Ultrafiltration experiments were also performed to evaluate the effects of sucrose/maltose on the filtrate flux. The presence of NPIs was identified by dynamic light scattering and SEM after filtration. Finally, different types of highly purified sucrose were examined.

## 2. Materials and Methods

### 2.1. Solution Preparation

A buffer solution of 20 mM histidine at pH 6 was prepared by dissolving the calculated amount of L-histidine and L-histidine HCl (Merck Millipore, Darmstadt, Germany) in deionized (DI) water supplied by a Millipore Milli-Q^®^ Direct 8 water purification system (Merck Millipore). Maltose (BioXtra, ≥99%, Merck Millipore, Darmstadt, Germany) and various types of sucrose (99.5% purified from Daejung Co., (Seoul, Korea); BioUltra^®^ (Vogen, Denmark), ACS reagent (St. Louis, MO, USA), Emprove^®^ Essential and Puriss^®^ from Merck Millipore (Darmstadt, Germany) were used. The pH of the solution was measured using a pH meter (Orion Versa Star Pro, Thermo Fisher Scientific, Waltham, MA, USA), and was adjusted by adding 0.2 N NaOH or 0.1 M HCl if necessary. Each buffer solution was used after vacuum filtration using a 0.2 μm hydrophilic PTFE membrane filter (SciLab, Seoul, Korea). Feed solutions for diafiltration were prepared with 7 g/L of human IgG, purchased from Merck Millipore (Darmstadt, Germany).

The viscosity of the solutions was measured using a stress-controlled rheometer (AR-G2, TA instrument, New Castle, DE, USA) with a 60 mm parallel plate at 23 °C. The average value from duplicated measurements collected at a shear rate of 100 s^−1^ was used. Note that the viscosity of 20 mM histidine with 100 mM sucrose (or maltose) was 1.3 mPa·s, whereas that of only the histidine buffer was 1.1 mPa·s.

### 2.2. Diafiltration Experiments

**Figure 1:**
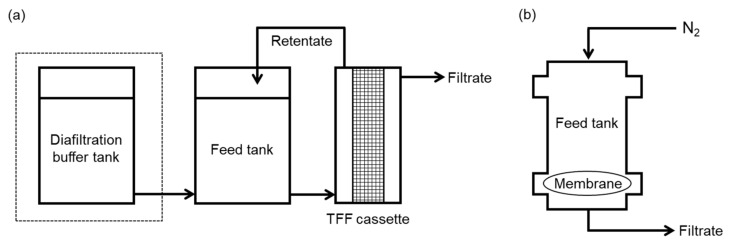
Schematic diagram of diafiltration and ultrafiltration using either (**a**) tangential flow filtration system or (**b**) normal flow filtration system.

Diafiltration (DF) experiments were performed using a tangential flow filtration (TFF) system, as shown is Figure 1a. Pellicon 3 TFF cassettes with D-screen and 30 kD Ultracel^®^ membranes (Merck Millipore, Darmstadt, Germany) were used. The cassettes were initially flushed with at least 500 mL of deionized (DI) water to remove any storage solution followed by conditioning with 20 mM histidine buffer for 20 min. Constant volume diafiltration was conducted at a flow rate of 51 mL/min (feed flux of 350 L/h/m^2^) using a pump (Masterflex, Gelsenkirchen, Germany), and at a transmembrane pressure (TMP) of 140 kPa (20 psi), by adjusting the pressure regulator on the retentate exit. Moreover, 50 mL of feed solution (7 g/L of IgG solution in 20 mM histidine buffer at pH 6) was used, and the histidine buffer with 100 mM sucrose (or maltose) was used as a DF buffer. Data relating to the filtrate flux were obtained by measuring the permeate mass, which was converted to volume using the density value for each buffer up until 10 diavolumes (i.e., 500 mL of filtrated volume). After the experiments, the cassettes were cleaned using 0.2 N NaOH for 30 min followed by flushing with 2 L of DI water.

### 2.3. Ultrafiltration Experiments

Ultrafiltration (UF) experiments were mostly performed using a normal flow filtration (NFF) system (HP4750, Steriltech, Auburn, WA, USA) with a disc type 30 kD Ultracel^®^ membrane (Merck Millipore, Darmstadt, Germany), as shown in Figure 1b. Limited UF experiments were carried out using a TFF system, without an input of DF buffer (dotted box in Figure 1a). The membranes were treated with isopropyl alcohol (IPA; Daejung Co., Seoul, Korea) for 15 min before use, in order to remove residual impurities and to get the membrane fully wet. Then, the membrane was equipped in the system, followed by flushing with 50 mL of DI water and conditioning with 15 mL of buffer. Furthermore, 280 mL of sucrose (or maltose) solution in the histidine buffer was used as a feed solution. UF experiments using a NFF system were conducted at a constant pressure of 100 kPa, at a stirring speed of 800 rpm. The filtrate flux was calculated by measuring the time taken to increase the weight of the permeate by 10 g.

After filtration, the membrane was carefully removed from the system to inspect the surface morphology by field emission scanning electron microscopy (JSM-6701F, JEOL, Tokyo, Japan). Additionally, the hydrodynamic diameter of sucrose in the feed/filtrate solution was analyzed using dynamic light scattering (Particulate systems, Nanoplus HD, Micromeritics, Norcross, GA, USA) with a scattering angle of 165°. Samples were placed in micro quartz cuvette and measured at least three times to ensure reproducibility. The sucrose concentrations were determined by the phenol-sulfuric method [20] using a microplate reader (SpectraMax M5, Molecular Devices, San Jose, CA, USA) at the wavelength of 490 nm. The absorbance of the samples was measured three times and the average value was used. The concentration of sucrose was analyzed against a standard curve that was obtained in advance.

## 3. Results and Discussion

### 3.1. Filtrate Flux Behaviors during Diafiltration

**Figure 2:**
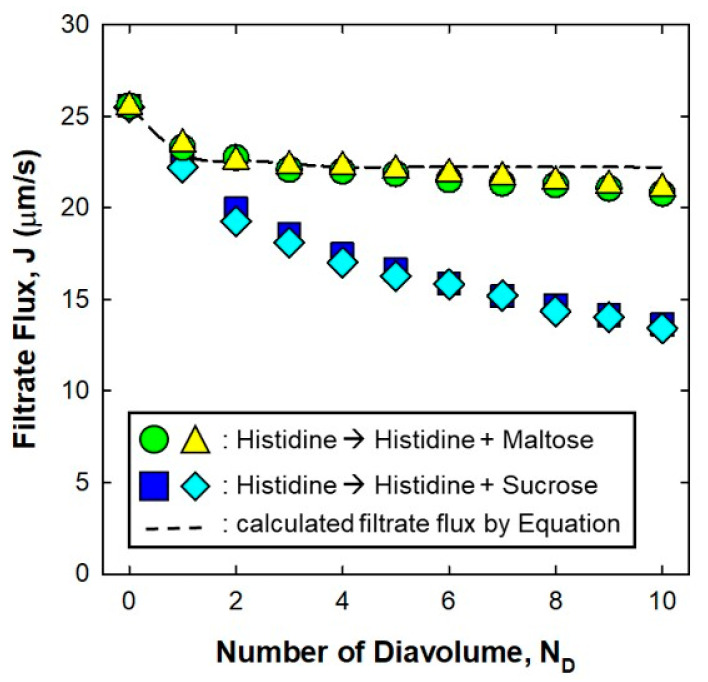
Experimental data for the filtrate flux as a function of the number of diavolumes (N_D_) during diafiltration at a feed flux of 350 L/h/m^2^ and a TMP of 140 kPa. The feed solution consisted of 7 g/L of IgG solution in 20 mM histidine buffer at pH 6. The diafiltration buffer was the feed solution that included 100 mM maltose or sucrose. The dashed curve represents the filtrate flux calculated by Equation (1).

Figure 2 shows the data for the filtrate flux as a function of the number of diavolumes during a set of diafiltration experiments using 7 g/L of IgG in 20 mM histidine buffer as a feed solution and the histidine buffer with 100 mM sucrose (99.5% purified from Daejung) solution as a diafiltration buffer. Data are shown for two repeat experiments, with the deviations between experiments typically less than 5% for any number of diavolumes. The filtrate fluxes in both solutions gradually decreased from 26 μm/s to 21 μm/s in maltose and to 14 μm/s in sucrose solution. The dashed curves in Figure 2 are the filtrate flux, calculated as:

(1) $$J_{\mathrm{cal}}=\frac{J_{0}\cdot \eta_{0}}{\eta }$$

where J_cal_ is the calculated filtrate flux at each diavolume, J_0_ is the measured initial flux, η_0_ is the solution viscosity (i.e., 1.1 mPa·s for 20 mM histidine buffer) and η is the solution viscosity at each diavolume, obtained from linear correlations between the sucrose/maltose concentration and the viscosity of the solution (e.g., 1.3 mPa·s for the histidine buffer with 100 mM sucrose or maltose). The calculated filtrate flux decreased slightly with higher diavolumes, of which the solution viscosity increased as the concentration of maltose or sucrose increased. Although the behavior of the filtrate flux of the histidine buffer with maltose was similar to that of the calculated filtrate flux, that of the histidine buffer with sucrose solution decreased significantly. This result is more appreciable than that of the previous study, which represented decreased water permeability of only ~12% after diafiltration with 60 g/L of mAb from 20 mM phosphate buffer with 58 mM NaCl at pH 7 to 20 mM histidine with 250 mM sucrose at pH 6.5 [21]. This discrepancy could be attributed to the use of different experimental conditions, such as protein and/or types of sucrose.

### 3.2. Filtrate Flux Behaviors during Ultrafiltration

**Figure 3:**
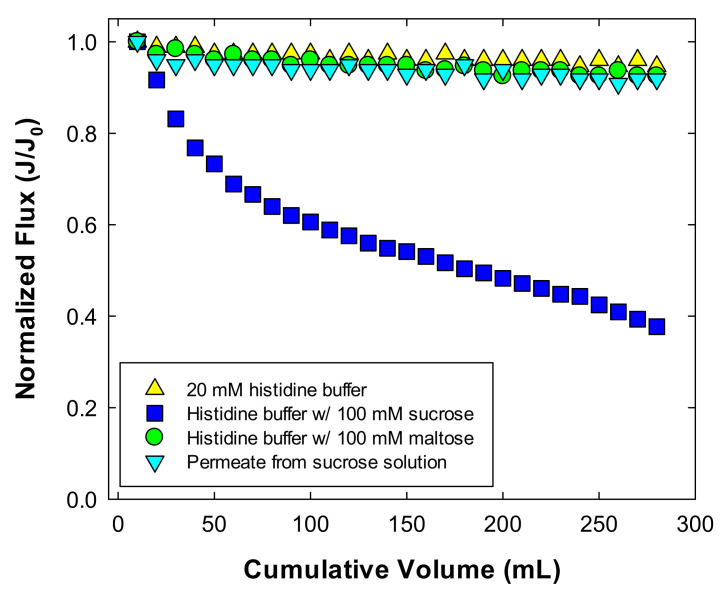
Normalized flux as a function of the filtrated volume during ultrafiltration using the normal flow filtration system at an applied pressure of 100 kPa, with a stirring speed of 800 rpm.

**Figure 4:**
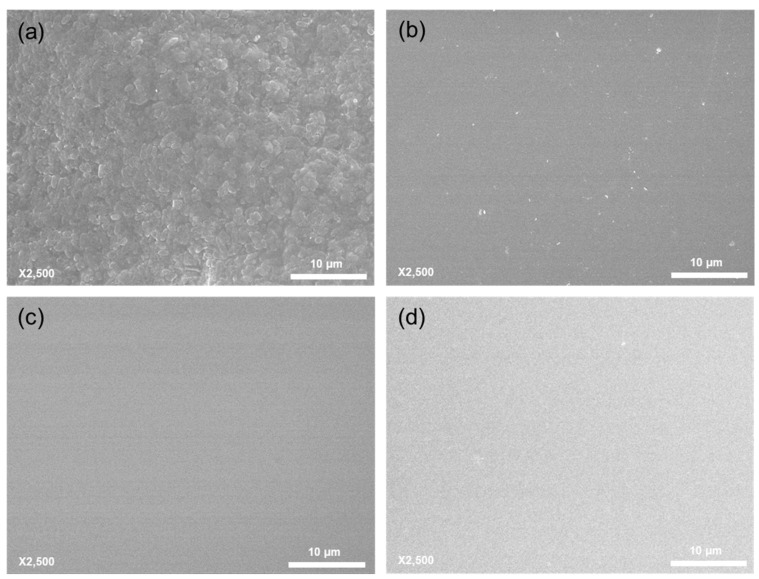
SEM images of the membrane surface after filtration using 20 mM histidine buffer with (**a**) 100 mM sucrose, (**b**) 100 mM maltose at pH 6, (**c**) permeate from sucrose solution and (**d**) control.

The effect of sucrose on the filtrate flux was confirmed by conducting a series of ultrafiltration experiments using a 30 kD Ultracel^®^ membrane in normal flow filtration mode. Figure 3 shows the normalized flux with 20 mM histidine buffer at pH 6, the buffer with 100 mM maltose or 100 mM sucrose, and the permeate obtained from the filtration of 100 mM sucrose solution. The filtrate flux with 20 mM histidine buffer was maintained as expected, and a similar result was obtained with the maltose solution (<10% decrease in the flux, which could be due to the pressure drop when performing normal flow filtration [22]). On the other hand, the filtrate flux of the sucrose solution significantly decreased to 38% of the initial flux. Note that the initial flux (J_0_) for both sucrose and maltose solutions was 85–90 μm/s, and that for the buffer only was approximately 100 μm/s. An ultrafiltration experiment with the permeate obtained from the filtration of 100 mM sucrose, revealed a significant decreased in the filtrate flux, consistent with the results obtained with the buffer only, or with the maltose solution. These results indicate that membrane fouling occurred with the sucrose solution. Observation of the membrane surface after filtration of sucrose solution using SEM (Figure 4) indicated that particulates (i.e., possible NPIs and sucrose) appeared to be stacked on the membrane surface as shown in Figure 4a, whereas the membrane filtrated with the permeate from sucrose solution and the control membrane were entirely free of particulate matter (Figure 4c,d). A few impurities were observed after filtration of maltose solution (Figure 4b), which insignificantly affected the filtrate flux. 

In addition, the concentration of sucrose from the permeate and residual feed solution was analyzed by the phenol-sulfuric method [20] when performing UF experiment using 20 mM histidine with 100 mM sucrose solution. As results, slightly decreased sucrose concentration (~94 mM) was observed in the permeate at 200 mL, whereas a similar concentration was observed in the residual feed solution. The results of lower concentration in the permeate indicated that some sucrose molecules were attached on the membrane. Adsorbed sucrose concentration analyzed after a detaching step (i.e., immersion in DI water, vortexing for 5 min, and ultrasonication for 5 min) appeared to be approximately 55 μg/cm^2^. Note that the concentration of sucrose or maltose from UF experiments using either the permeate from sucrose solution or 20 mM histidine with 100 mM maltose solution was almost equal to the feed concentration (100 mM).

**Figure 5:**
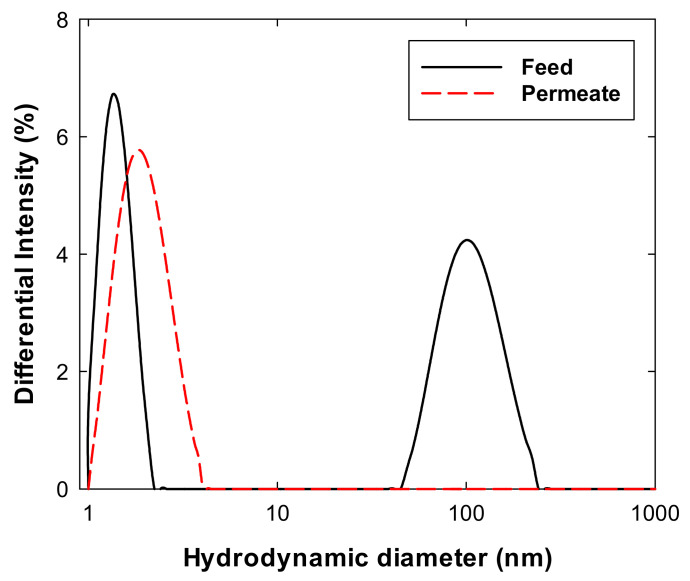
Hydrodynamic diameter of sucrose measured using dynamic light scattering. Feed was prepared as 20 mM histidine with 100 mM sucrose solution at pH 6 and the permeate was collected after filtration experiment.

Figure 5 shows the dynamic light scattering results for 20 mM histidine with 100 mM sucrose at pH 6 (black line) and permeate (red dashed line) from the ultrafiltration experiment with histidine with sucrose solution. The permeate solution only exhibited one peak at 1–2 nm, whereas two peaks were detected for the feed solution, with one peak for the sucrose, and another peak at approximately 110 nm. The second of these peaks indicates the nanoparticulate impurities (NPIs) observed in the previous studies [18,19]. These impurities are mostly composed of ash and/or high molecular weight dextran, possibly affecting the protein stability in the formulation process. Therefore, the particulates shown in Figure 4a are partly large NPIs and partly sucrose to which NPIs adhered. It is noted that the hydrodynamic diameter of maltose on both feed and permeate was only observed at 1–2 nm, as the results with the permeate of sucrose solution (Figure S1). 

**Figure 6:**
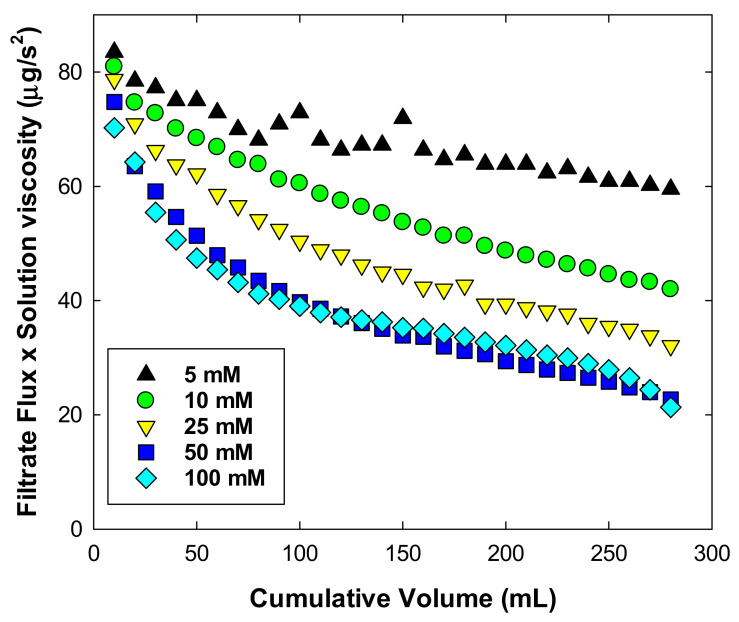
Normalized flux as a function of the filtrated volume during ultrafiltration using a normal flow filtration system at an applied pressure of 100 kPa, with a stirring speed of 800 rpm.

Figure 6 shows the filtrate flux behavior on sucrose concentration using an NFF system. Data were obtained with 5–100 mM sucrose in 20 mM histidine buffer at pH 6. Filtrate flux was calculated by multiplying the solution viscosity by each filtrate flux, in order to exclude the effect of the viscosity of the solution on the filtrate flux. As shown in Figure 6, more flux decline occurred as sucrose concentration increased from 5 mM to 50 mM, and similar flux behavior was observed between 50 mM and 100 mM sucrose solutions. This result indicates that more NPIs were included as higher concentrations of sucrose, resulting in more membrane fouling. The content of NPIs included in 50 mM sucrose solution might be a maximum affected to flux behavior in the condition of this study. 

**Figure 7:**
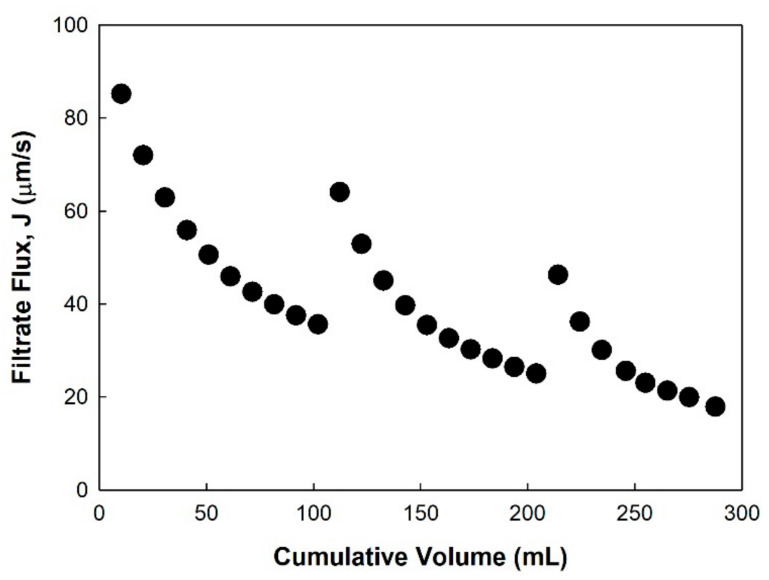
Filtrate flux behavior of pressure release during ultrafiltration using the normal flow filtration system with 20 mM histidine at pH 6 with 100 mM sucrose solution. The applied pressure was 100 kPa and the stirring speed was 800 rpm. Pressure release was carried out for 10 min at approximately 100 and 200 mL of the cumulative volume.

An additional ultrafiltration experiment was performed similarly to the normal flow filtration, but releasing the pressure during the filtration at 100 mL and 200 mL of filtrated volume for 10 min each. The behavior of the filtrate flux, shown in Figure 7, is described as follows. The initial filtrate flux decreased from 85 μm/s to 36 μm/s after the filtration of 100 mL of sucrose solution. After releasing the pressure for 10 min, the filtrate flux abruptly increased to 64 μm/s with pressurizing, and then decreased to 25 μm/s. Additional release and application of pressure resulted in similar behavior (i.e., 46 μm/s → 18 μm/s). The flux recovery was calculated by taking into account the extent to which the flux decreased and recovered was approximately 55% at each of the pressure releasing steps. The filtrate flux is lowered as a consequence of concentration polarization and fouling [23]. The effect of concentration polarization on the flux reduction could be shown to be 55% of the flux recovery, of which the effect was temporarily excluded by stirring without any permeate drag force. It also indicates that approximately 45% of the flux reduction occurred because of membrane fouling with NPIs and sucrose (Figure 4a and Figure 5).

### 3.3. Effects of Sucrose Types on Filtrate Flux 

**Figure 8:**
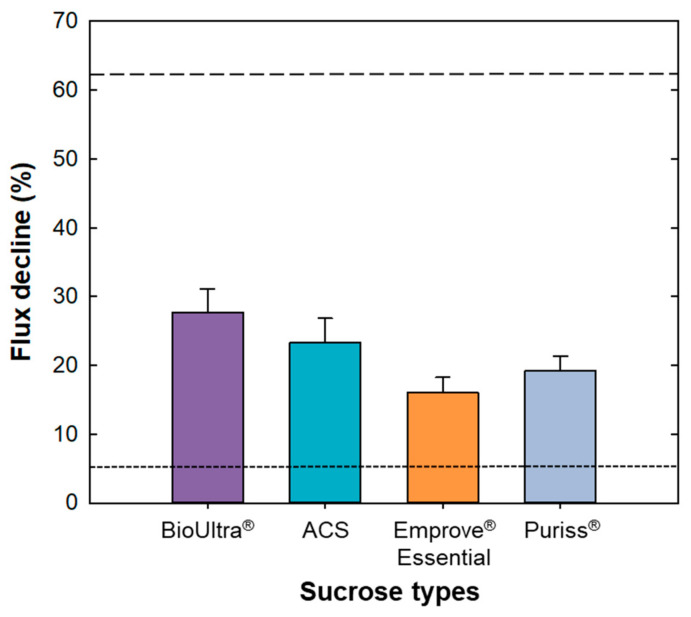
Flux decline using the normal flow filtration system with different types of sucrose (20 mM histidine at pH 6 with 100 mM sucrose solution). The upper dashed line indicates the flux decline using the sucrose with impurities and the dotted line at the bottom indicates the flux decline, with only buffer owing to the pressure drop.

Figure 8 shows the percentage of flux decline when filtrate experiments were performed using a normal flow filtration system with various types of sucrose solutions. Data are shown for the results with 20 mM histidine at pH 6 with four types of 100 mM sucrose: BioUltra^®^ (for molecular biology, ≥99.5%), ACS reagent, Emprove^®^ Essential (high-quality products for biopharmaceutical formulation) and Puriss^®^ (analytical specification of Ph. Eur., BP, NF). Note that the last two types of sucrose recently became available. As shown in Figure 8, the initial flux decreased by 27.7 ± 3.4%, 23.3 ± 3.5%, 16.0 ± 2.2%, and 19.2 ± 2.1% for BioUltra^®^, ACS reagent, Emprove^®^ Essential, and Puriss^®^ after the filtration of 280 mL at 100 kPa of applied pressure with a stirring speed of 800 rpm. The upper dashed line indicates the flux decline of the sucrose solution shown in Figure 3 (62.3%) and the dotted line at the bottom represents the results for the buffer only (5.3%). 

The flux decline of the sucrose solution shown in Figure 3 is much higher because of the presence of NPIs and sucrose on the membrane, as observed in the SEM image (Figure 4a), and determined with DLS analysis (Figure 5) and sucrose quantification analysis. On the other hand, the flux decline of the other high-purity sucrose solutions from Merck Millipore was significantly lower at 16–28%. The filtrate flux using Emprove^®^ Essential showed the lowest decline in the order of Puriss^®^, ACS reagent and BioUltra^®^. It is noted that NPIs were not observed in the SEM image after filtration and DLS analysis (data not shown). Limited UF experiments were carried out using the TFF system because the UF/DF process is performed using the TFF system [8]. Moreover, 30 kD Ultracel^®^ cassette membranes with D screen (Merck Millipore) were used, and UF experiments were operated at a 45 mL/min of flow rate and 100 kPa of TMP. Data obtained with 100 mM of 99.5% purified sucrose from Daejung Co. or Emprove^®^ Essential in the histidine buffer as a feed solution. Filtrate flux of Emprove^®^ Essential was almost maintained (<5% flux decline), whereas that of purified sucrose decreased 12% approximately for 30 min of operation time (Figure S2). Additionally, decreased filtrate flux was barely recovered even after the cleaning, indicating that membrane fouling via NPIs from sucrose was irreversible. Therefore, Emprove^®^ Essential, which resulted in the smallest flux decline, is suitable for biopharmaceutical formulation.

## 4. Conclusions

This study evaluated the filtrate flux behavior of sucrose in solution, which is often used as an excipient for the formulation of biotherapeutics. Filtrate flux data were obtained by conducting diafiltration experiments using a tangential flow filtration system and ultrafiltration experiments using a normal flow filtration system. In the diafiltration experiments, the filtrate flux of the histidine buffer with sucrose (of which the purity was relatively lower than in subsequent experiments) solution significantly decreased compared with that of the maltose solution. This large decline in the flux was also observed when the histidine buffer with sucrose solution was used in a normal flow filtration system. As was previously reported [18,19], nanoparticulate impurities (NPIs) existed in the sucrose solution and were observed in the SEM image after normal flow filtration experiments (Figure 4), and in the form of an intense (~110 nm) peak on the DLS size distribution graph (Figure 5). Additionally, some sucrose molecules were also adsorbed onto the membrane surface (~55 mg/cm^2^), as determined by the phenol-sulfuric method. The decreased filtrate flux owing to NPIs and sucrose could be explained by concentration polarization and membrane fouling phenomena.

Highly purified sucrose, especially Emprove^®^ Essential, resulted in a much smaller flux decline (~16%) than that of sucrose, including NPIs (~62%), because of the absence of NPIs. Additionally, negligible flux decline was observed with the tangential flow filtration system, which is usually used to perform UF/DF. These results provide important insights into the factors governing the filtrate flux behavior in the UF/DF process using appropriate excipients for the formulation of biotherapeutics.

## Data Availability

Not applicable.

## Supplementary Materials

The following are available online at https://www.mdpi.com/article/10.3390/membranes11100775/s1, Figure S1: Hydrodynamic diameter of maltose measured using dynamic light scattering. Feed was prepared as 20 mM histidine with 100 mM maltose solution at pH 6 and permeate was collected after filtration experiment. Figure S2: Normalized flux as a function of operation time during ultrafiltration experiment using the tangential flow filtration system at a flow rate of 45 mL and a TMP of 100 kPa. Feed solution was 20 mM histidine with 100 mM sucrose solution at pH 6. Two types of sucrose were use; Emprove^®^ Essential which is high-quality products for biopharmaceutical formulation (black circle) and 99.5% purified sucrose (yellow triangle).
