# Supplementary material for: Effects of Impurities from Sugar Excipient on Filtrate Flux during Ultrafiltration and Diafiltration Process
Source: Membranes (Basel). 2021 Oct 11;11(10):775. doi: 10.3390/membranes11100775 (PMC8541299; doi:10.3390/membranes11100775)
Supplement: Supplementary file 1 [file membranes-11-00775-s001.zip › membranes-1381732-supplementary.pdf]

membranes

MDPI

Supplementary Material

Effects of Impurities from Sugar Excipient on Filtrate Flux during Ultrafiltration and Diafiltration Process

Jieun Lee, Jiwon Na and Youngbin Baek *

Department of Biotechnology, Sungshin Women's University, Seoul 01133, Korea; je_0229@nate.com (J.L.); qas111026@gmail.com (J.N.)
* Correspondence: ybbaek@sungshin.ac.kr

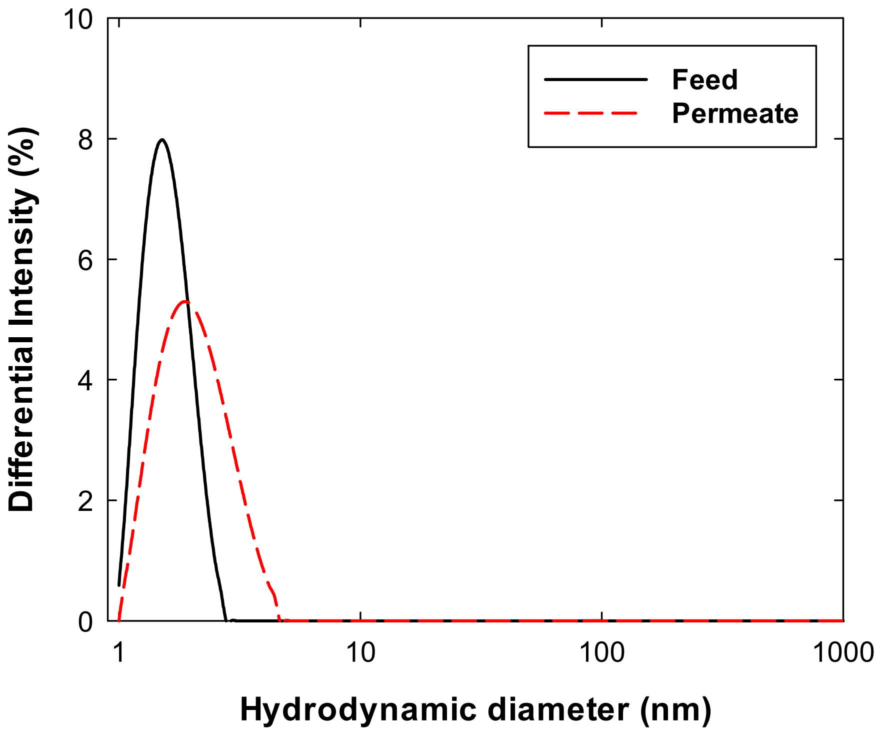

Figure S1. Hydrodynamic diameter of maltose measured using dynamic light scattering. Feed was prepared as 20 mM histidine with 100 mM maltose solution at pH 6 and permeate was collected after filtration experiment.

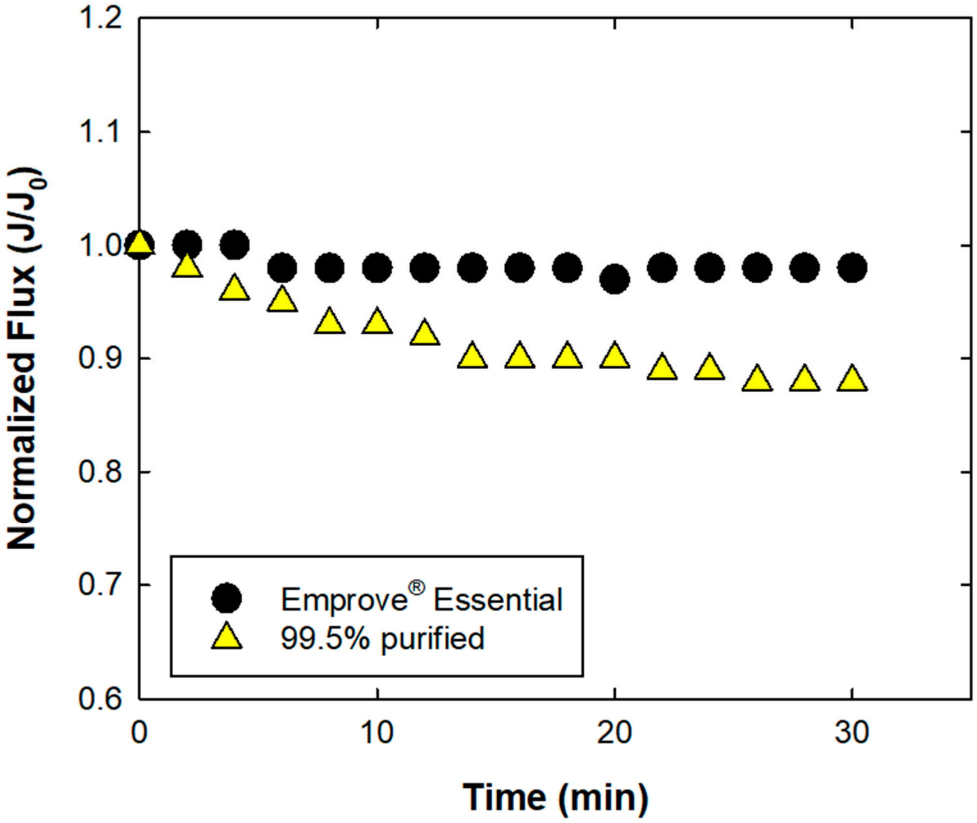

Figure S2. Normalized flux as a function of operation time during ultrafiltration experiment using a tangential flow filtration system at a flow rate of 45 mL/min and a TMP of 100 kPa. Feed solution was 20 mM histidine with 100 mM sucrose solution at pH 6. Two types of sucrose were use; Emprove® Essential which is high-quality products for biopharmaceutical formulation (black circle) and 99.5% purified sucrose (yellow triangle).
